# A distance geometry-based description and validation of protein main-chain conformation

**DOI:** 10.1107/S2052252517008466

**Published:** 2017-08-08

**Authors:** Joana Pereira, Victor S. Lamzin

**Affiliations:** a European Molecular Biology Laboratory, c/o DESY, Notkestrasse 85, 22607 Hamburg, Germany

**Keywords:** Ramachandran plot, protein stereochemistry, validation, geometrical strain, dipeptide unit, distance matrix, Euclidean orthogonal three-dimensional space, trypsin proteases

## Abstract

Understanding the protein main-chain conformational space forms the basis for the modelling of protein structures and for the validation of models derived from structural biology techniques. Presented here is a novel idea for a three-dimensional distance geometry-based metric to account for the fine details of protein backbone conformations. The metrics are computed for dipeptide units, defined as blocks of C^α^_*i*−1_—O_*i*−1_—C^α^_*i*_—O_*i*_—C^α^_*i*+1_ atoms, by obtaining the eigenvalues of their Euclidean distance matrices. These were computed for ∼1.3 million dipeptide units collected from nonredundant good-quality structures in the Protein Data Bank and subjected to principal component analysis. The resulting new Euclidean orthogonal three-dimensional space (DipSpace) allows a probabilistic description of protein backbone geometry. The three axes of the DipSpace describe the local extension of the dipeptide unit structure, its twist and its bend. By using a higher-dimensional metric, the method is efficient for the identification of C^α^ atoms in an unlikely or unusual geometrical environment, and its use for both local and overall validation of protein models is demonstrated. It is also shown, for the example of trypsin proteases, that the detection of unusual conformations that are conserved among the structures of this protein family may indicate geometrically strained residues of potentially functional importance.

## Abstract 2

The conformation of the protein main chain is described in a novel three-dimensional space derived from the interatomic distances. This allows the local and overall validation of protein backbone geometry and the detection of residues that are strained for reasons of their function.

## 1. Introduction   

**Figure 1:**
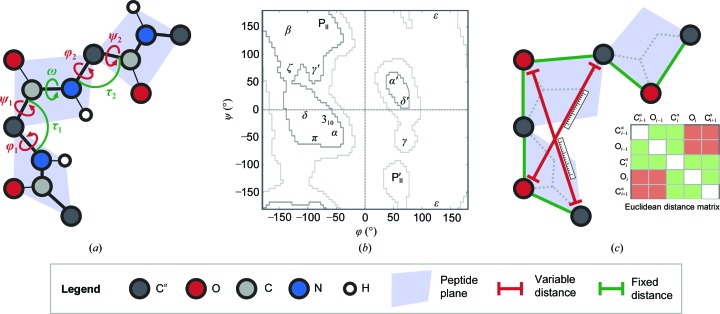
Protein backbone. (*a*) Full-atom representation described by the Ramachandran φ and ψ angles (in red); the ω torsion and τ stretching angles are also shown (in green). (*b*) The joint distribution of the Ramachandran φ and ψ angles with the allowed (light grey) and favoured (dark grey) regions according to Lovell *et al.* (2003 ▸); the nomenclature of different regions is according to Hollingsworth & Karplus (2010 ▸). (*c*) Five-atom (double-plane) representation with the conformationally variable interatomic distances shown in red; the distance-geometry based concept used in this work is depicted by a 5 × 5 interatomic distance matrix.

Knowledge of the structures of biological macromolecules is imperative for the understanding of their function in cellular processes and their role in human diseases. Deciphering and validating these structures is essential for biological research. Protein structures are formed by sequences of amino acids condensed through peptide bonds into a universe of conformations. When searching for a convenient notation for polypeptide conformation, Ramachandran and coworkers suggested the use of two main-chain torsion angles, φ (C_*i*−1_—N_*i*_—C^α^_*i*_—C_*i*_) and ψ (N_*i*_—C^α^_*i*_—C_*i*_—N_*i*+1_) (Ramachandran *et al.*, 1963 ▸; Fig. 1 ▸ *a*). With the emergence of software such as *PROCHECK* (Laskowski *et al.*, 1993 ▸) and *MolProbity* (Chen *et al.*, 2010 ▸), enabling parts of the model located in allowed or disallowed regions of the Ramachandran plot to be indicated ‘on the fly’, the Ramachandran plot (Fig. 1 ▸ *b*) has become one of the most important main-chain quality indicators for a protein model (Lovell *et al.*, 2003 ▸; Read *et al.*, 2011 ▸; Carugo & Djinović-Carugo, 2013 ▸).

The joint use of torsion angles has formed the basis for the development of other tools for the description and validation of protein conformation. Examples include the description of different turns (Oldfield & Hubbard, 1994 ▸), the validation of C^α^-only models (Kleywegt, 1997 ▸) and the description of protein backbone conformation with respect to the location of C^α^ atoms (Peng *et al.*, 2014 ▸) or to the formation of hydrogen bonds (Penner *et al.*, 2014 ▸).

A two-dimensional description of the polypeptide conformational space by the Ramachandran dihedral angles is however a simplification and does not fully account for the natural variation in the interatomic and angle-bonded distances of the protein backbone (Engh & Huber, 1991 ▸, 2006 ▸). It also hides information about the stretched geometry around the C^α^_*i*_ atom (Malathy Sony *et al.*, 2006 ▸; Berkholz *et al.*, 2009 ▸; Touw & Vriend, 2010 ▸). In refined protein structures the stretching angle τ (N_*i*_—C^α^_*i*_—C_*i*_; Fig. 1 ▸ *a*) varies from 107.5 to 114.0° (Berkholz *et al.*, 2009 ▸). Therefore, validation methods such as *WHAT_CHECK* (Hooft *et al.*, 1996 ▸) and *MolProbity* (Chen *et al.*, 2010 ▸) examine the values of φ, ψ and τ using a combination of different tools.

The apparent planarity of the *trans* peptide unit arises from the partial double-bonded character of the peptide bond, which forces the ω (C^α^_*i*_—C_*i*_—N_*i*+1_—C^α^_*i*+1_) torsion angle (Fig. 1 ▸ *a*) to be around 180° (MacArthur & Thornton, 1996 ▸). The polypeptide chain can then be regarded as a set of peptide planes connected at the C^α^ positions. As three non-collinear points are sufficient to define a plane, in principle any three atoms within the peptide unit can be used. However, given that the C^α^_*i*_—C_*i*_—N_*i*_—C^α^_*i*+1_ atoms in a *trans* peptide lie almost on a straight line (Fig. 1 ▸ *a*), the most remote C^α^_*i*_, O_*i*_ and C^α^_*i*+1_ atoms in the peptide plane are the best three points to define it (Fig. 1 ▸ *c*). With this, we define a double-plane dipeptide unit, C^α^_*i*–1_—O_*i*−1_—C^α^_*i*_—O_*i*_—C^α^_*i*+1_, around each C^α^_*i*_ position.

As molecular conformation can be defined by the relative position of atoms and by the chirality of asymmetric atomic groups (Fig. 1 ▸ *c*; Crippen & Havel, 1988 ▸; Leach, 1991 ▸), we propose a new look at a protein backbone conformation by considering the interatomic distances within these blocks of five atoms. We show that such an approach allows an orthogonal three-dimensional conformational space and demonstrate its use for the description of protein polypeptide conformation. The proposed description accounts for all conformations that a dipeptide unit adopts in protein structures and is able to indicate C^α^ atoms that are in an unlikely or unusual geometrical environment. In addition, the higher dimensionality of this conformational space makes it inherently more informative than, for example, the two-dimensional Ramachandran plot. Here, we present an application of the developed approach for both local and global validation of protein backbone and for the analysis of conserved geometrical strains using the structures of the trypsin protein family as an example.

## 2. Materials and methods   

### 2.1. Collection of the dipeptide units   

A set of dipeptide units representing the conformations present in the Protein Data Bank (PDB) was collected as follows. Protein chains were taken from the PDBe (Velankar *et al.*, 2010 ▸; as of 30 September 2014) with a pairwise sequence identity below 50% using the PDB50 clusters (Li & Godzik, 2006 ▸). Selected structures were obtained using X-ray crystallography at a resolution of better than 2.5 Å with a crystallo­graphic *R* factor of below 25%, an *R*_free_ − *R* factor difference of below 5% and with PDB validation report clashscore and Ramachandran outliers percentiles (Read *et al.*, 2011 ▸) of better than 40%. A total of 4862 chains were selected, with *R*-factor and *R*_free_ distributions fairly representing the PDB content with some outliers removed. Each selected protein chain was broken into five-atom dipeptide units, and only those comprising main-chain C^α^ and O atoms with unit occupancy and atomic displacement parameters below 80.0 Å^2^ were taken.

In order to further exclude dipeptide units representing unlikely or problematic backbone regions (outliers), two rounds of filtering were applied based on the interatomic distances: (i) for the distributions of the ‘fixed distances’ between atoms in the same peptide unit, a Gaussian mixture analysis was performed using the *normalmixEM* function from the *mixtools* R package (Benaglia *et al.*, 2009 ▸) and only dipeptide units composed of *trans* peptide planes with all fixed distances within the 3σ interval of the broader Gaussian distribution in the mixture model analysis (Supplementary Fig. S1 and Table S1) were accepted, and (ii) for the distributions of the ‘variable distances’ between atoms in different peptide units, the interval comprising 99.8% of the dipeptide set was determined using the highest density region method as implemented in the *hdrcde* R package (Hyndman, 1996 ▸; Samworth & Wand, 2010 ▸) and only dipeptide units within these intervals (Supplementary Fig. S2) were accepted. A total of 1 360 370 dipeptide units were selected with a median τ of 111.3 ± 2.3°.

For each collected chain, its fold class was assigned using the SCOPe database (Fox *et al.*, 2014 ▸) and its local secondary-structural information was obtained using *DSSP* (Kabsch & Sander, 1983 ▸; Touw *et al.*, 2015 ▸). For each dipeptide unit, the secondary-structural class was assigned to the residue represented by the central C^α^_*i*_ atom. The class for the preceding C^α^_*i*−1_ atom was also stored, and a dipeptide unit was marked to belong to a secondary-structural element only if both of these residues were assigned to the same class. Although the *DSSP* annotation may depend on the accuracy of the local geometry (Kabsch & Sander, 1983 ▸; Martin *et al.*, 2005 ▸; Zhang & Sagui, 2015 ▸), the use of dipeptides for construction of the DipSpace is not dependent on the secondary-structure assignment.

The three axes of inertia and the radius of gyration for each dipeptide unit were obtained by eigendecomposition of its 3 × 3 variance–covariance coordinate matrix (Elias, 1977 ▸).

### 2.2. Transformation to the DipSpace   

For each dipeptide unit, a 5 × 5 Euclidean distance-squared matrix was computed. This matrix has five zero main diagonal and ten unique positive off-diagonal entries: six corresponding to the fixed distances and four to the variable distances (Fig. 1 ▸ *c*). Such matrices have one positive and four negative or zero eigenvalues (Marcus & Smith, 1989 ▸). Since the sum of these eigenvalues is equal to zero, the information on the distances in a five-atom dipeptide unit is contained in the four negative eigenvalues (Supplementary Fig. S3). We refer to these, with their signs changed, as λ_1_ > λ_2_ > λ_3_ > λ_4_. These eigenvalues were computed for each dipeptide unit and their square root was taken, setting their magnitudes on an angstrom scale. These, for all collected dipeptide units, were then subjected to principal component analysis (PCA; Wold *et al.*, 1987 ▸). This resulted in three decorrelated principal components which describe the axes of the new protein backbone conformational space: the DipSpace. For a given dipeptide unit, its coordinates in the DipSpace can be obtained as described in Appendix *A* [App appa].

Since mirror-imaged dipeptide units share the same distance information (Crippen & Havel, 1988 ▸), the DipSpace was divided into two chiral subspaces. Although the five-atom dipeptide units have two asymmetric points, only the sign of one of them is needed, as the information about the other is embedded in the distances (Crippen & Havel, 1988 ▸). We define the dipeptide chirality as the sign of the chiral volume (Leach, 1991 ▸) made by the C^α^_*i*−1_, O_*i*−1_, C^α^_*i*_ and O_*i*_ atoms. Dipeptides with negative chirality build up the ‘negative subspace’ and those with positive chirality build up the ‘positive subspace’. The negative subspace is more populated, representing the conformational preferences of the protein backbone.

### 2.3. Conformational description by the DipSpace axes   

We selected five conformationally representative dipeptide units from the negative (more populated) subspace that were approximately equally separated along each DipSpace axis. They also represent a route connecting highly populated regions in DipSpace and, at the same time, show a continuous path when projected on the Ramachandran plot. For the path along the pc1 axis, the pc2 and pc3 coordinates were kept at about 0.7 and 0.3, respectively. For the path along pc2, both pc1 and pc3 were set to zero. For the path along the pc3 axis, the pc1 and pc2 coordinates were kept at −0.7 and −0.2, respectively. Movies (Supplementary Videos S1, S2 and S3) demonstrating the conformational variation of dipeptide units along these directions in the DipSpace were generated using *PyMol* (DeLano, 2002 ▸).

### 2.4. Calculation of the DipScore   

As the DipSpace was built to reflect the occurrence of the conformations present in the PDB (‘the success cases’), we additionally require ‘the failure cases’ in order to compute DipScores and to put the method on a probabilistic basis. Accordingly, we constructed a randomly sampled ‘noise’ model, representing a probability density function of an event occurring at random, composed of 1 200 000 ‘dipeptides’ obtained by the random placement of five points inside a sphere of 4.0 Å radius (Supplementary Fig. S4) with no additional conditions applied. Indeed, any restrained noise model would bias the DipScores towards our belief of what the restraints should be. For each of these random placements, their distance matrices and eigenvalues were computed and then transferred to the DipSpace by applying the transformation given in Appendix *A* [App appa]. The obtained random-noise model is not biased to any stereochemistry and reflects both plausible and impossible conformational arrangements.

The DipSpace was binned on a three-dimensional grid spanning −1.975 through 1.975 Å with a step of 0.05 Å, containing a total of 512 000 grids. The value for each grid was assigned to the number of points (dipeptide conformations) located within an empirically defined radius of 0.09 Å, normalized by the total number of points in the subspace. The density of the PDB-derived points (*d*_PDB_) was determined from either the negative or the positive subspace, following the chirality of the dipeptide unit. The same procedure was carried out for the randomly generated ‘dipeptides’, resulting in the density of the noise model (*d*_random_), which was the same for both subspaces. We note that the density of the noise model is defined up to a multiplicative constant of proportionality, which can be set to 1 without loss of generality and without a change in the information content of the noise model. Therefore, for each DipSpace grid, the DipScore was computed using 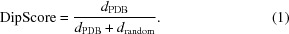
For a given dipeptide unit, its DipScore was calculated by computing its DipSpace coordinate in the corresponding subspace and applying a parabolic 3 × 3 × 3 three-dimensional interpolation (Press *et al.*, 1999 ▸) between the surrounding DipSpace grids. The numerical data for the DipSpace are provided in the Supporting Information.

In order to define the boundaries for favoured, allowed, generously allowed and disallowed DipScore values, the cumulative density distribution of the DipScores computed for all points in the DipSpace was used. Building on a classification suggested for the Ramachandran plot by Lovell *et al.* (2003 ▸), a favoured DipScore region corresponds to the top 98% of the data (*i.e.* all DipScores above percentile 2.0), an allowed region to 99.8% of the data (DipScore percentiles between 2.0 and 0.2) and a generously allowed region to 99.95% of the data (DipScore percentiles between 0.2 and 0.05). Dipeptide units with a DipScore lower than that for the generously allowed region (the remaining 0.05% of the data) were then classified as disallowed or outliers.

### 2.5. Calculation of χ_score_   

The distribution of the DipScores computed for each C^α^ atom provides important information about the overall stereochemical consistency of a given protein model. It would be expected that each of the first four central moments of the DipScore distribution – the mean (*m*_1_), variance (*m*_2_), skewness (*m*_3_) and kurtosis (*m*_4_) – computed for a set of good models would follow a Gaussian distribution, thus allowing the calculation of four *Z*-scores (*Z_i_*) using
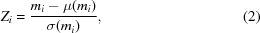
where μ(*m_i_*) is the mean and σ(*m_i_*) is the standard deviation for each moment *m_i_*, within the set of good models.

To prove the Gaussian distribution of these central moments (Supplementary Fig. S5) and to estimate the values of μ(*m_i_*) and σ(*m_i_*), 538 protein chains of longer than 50 residues were randomly selected from the set of chains collected from the PDB. The DipScores for each residue and the first four central moments of their distribution were calculated. The median and the median absolute deviation (MAD_e_) were then used to estimate the population mean and standard deviation, respectively. 22 chains with at least one outlier moment (those with a value more than 4.0 MAD_e_ away from the median) were excluded. The mean (μ_*i*_) and the standard deviation (σ_*i*_) for the four moments (*m_i_*) of the remaining 516 chains (Supplementary Table S2) were used to calculate the *Z*-scores using equation (2). PCA was carried out over the *Z*-scores data set in order to decorrelate and combine them into a single-parameter scoring function, χ_score_ (Appendix *B* [App appb]). The favoured (98%), allowed (99.8%) and generously allowed (99.95%) regions for the χ_score_ function were computed similarly to those for the DipScore.

### 2.6. The protein test cases   

To test the developed method for model validation, the coordinates of four test cases representing different scenarios in protein structural analysis (PDB entries 1lml, 1n7s, 1qjp and 2fdq; Schlagenhauf *et al.*, 1998 ▸; Ernst & Brunger, 2003 ▸; Pautsch & Schulz, 2000 ▸; Costabel *et al.*, 2006 ▸) were taken from the PDBe. The experimental data for entry 1lml were downloaded from the Uppsala Electron Density Server (EDS; Kleywegt *et al.*, 2004 ▸) and the model was re-refined using *REFMAC*5 (Murshudov *et al.*, 2011 ▸). The *PDB_REDO* report for the 2fdq model and the coordinates of the rebuilt structure were obtained from the *PDB_REDO* databank (http://www.cmbi.ru.nl/pdb_redo/; Joosten *et al.*, 2009 ▸, 2014 ▸; Touw *et al.*, 2015 ▸). The *WHAT_CHECK* (Hooft *et al.*, 1996 ▸) and PDB validation (Read *et al.*, 2011 ▸) reports for each model were obtained from the PDBe. The number of nonglycine/non­proline Ramachandran plot outliers were computed using *MolProbity* (Chen *et al.*, 2010 ▸).

To test whether the developed method is able to identify geometrically strained residues (Karplus, 1996 ▸) that may not be seen in the Ramachandran plot, and to identify residues which are strained for possible functional reasons, we used the trypsin protein family as an example. Models were selected from the PDBe using the following criteria: a macromolecular name annotated as ‘trypsin’, a model consisting of one chain only, of longer than 200 residues, obtained using X-ray crystallography, and a favoured χ_score_ (computed according to Appendices *A* [App appa] and *B* [App appb]). This resulted in a total of 350 structures (Supplementary Table S7). Given the conservation of the trypsin fold (Rypniewski *et al.*, 1994 ▸; Perona & Craik, 1997 ▸), all models were superimposed on the model of porcine trypsin (PDB entry 2a31; Transue *et al.*, 2006 ▸) using the default settings of the *Chimera MatchMaker* function (Pettersen *et al.*, 2004 ▸). The Needleman–Wunsch algorithm (Needleman & Wunsch, 1970 ▸) was used with the BLOSUM62 matrix (Henikoff & Henikoff, 1992 ▸), a gap-extension penalty of 1 and secondary-structure information. The superposition was performed iteratively by the identification of C^α^–C^α^ pairs at distances of less than 2.0 Å. The obtained alignment was then used to find the correspondences between the porcine trypsin structure and the remaining 349 models for all C^α^–C^α^ pairs at a distance of less than 2.5 Å. The annotation of catalytic residues was taken from the Catalytic Site Atlas (CSA) database (Furnham *et al.*, 2014 ▸).

## 3. Results and discussion   

### 3.1. The distances in the sampled dipeptide units   

The interatomic distances in a dipeptide unit carry different geometrical and conformational information around a given C^α^ position. The six distances between atoms within the same peptide planes reflect the coordinate error and the tightness of the restraints applied during structure determination, but also the geometry and isomerization state of the peptide bond (Supplementary Fig. S1). They are not expected to vary considerably from their target values and henceforth are defined as ‘fixed’. The distribution of each of the ‘fixed distances’ in *trans* peptide units can be described by two Gaussian functions (Supplementary Fig. S1*c* and Table S1) having the same mean but different standard deviations. The minor component is about twice as broad. This suggests the presence of two types of *trans* peptide-unit populations, possibly arising from different weights applied to the geo­metrical restraints or from the different refinement strategies employed. The four ‘variable distances’ between atoms in different peptide planes (Fig. 1 ▸ *c*) reflect the conformation of the dipeptide unit, and their distribution is multimodal and asymmetric (Supplementary Figs. S2*a* and S2*b*).

### 3.2. The eigenvalues of the interatomic distance matrices and the DipSpace   

The distributions of the four eigenvalues (λ_1_ > λ_2_ > λ_3_ > λ_4_) calculated from the distance matrices have some resemblance to the distributions of the variable distances (Supplementary Fig. S2*b* and Table S3*a*). Only λ_1_ correlates strongly with the first principal moment of inertia of a dipeptide unit and the squared radius of gyration *R*_g_^2^. Its square root correlates with the O_*i*−1_—C^α^_*i*+1_ distance (*r* = 1.000, 0.980, 0.958, respectively). λ_2_ correlates with the second principal moment of inertia and its square root with the C^α^_*i*−1_—C^α^_*i*+1_ distance (*r* = 0.941 and −0.905; Supplementary Table S3).

**Figure 2:**
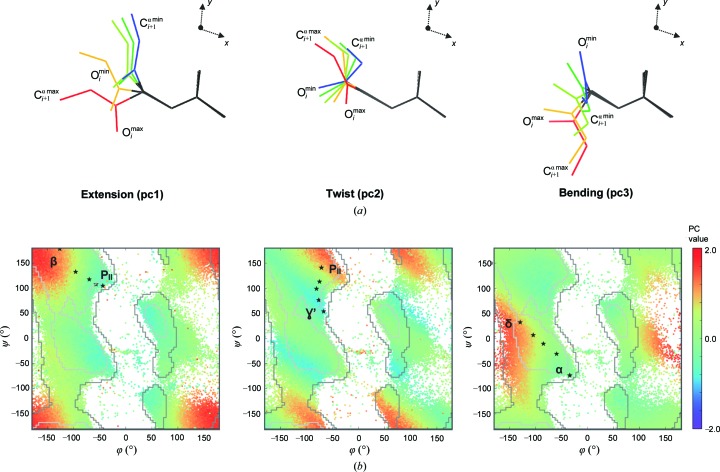
Conformations of a dipeptide unit described by the three DipSpace axes. (*a*) Representative dipeptide units from the negative subspace with their two DipSpace coordinates fixed while varying the third coordinate between its minimum (blue) and maximum (red) values, as described in §[Sec sec2]2. (*b*) Exemplary projection of the DipSpace on the Ramachandran plot with its general limits (Lovell *et al.*, 2003 ▸) shown. Stars mark the path through the conformations shown in (*a*). The nomenclature follows that of Hollingsworth & Karplus (2010 ▸): β, β-strands; α, α-helices, γ’, γ’-turns; δ, bridge region, several types of turns; P_II_, P_II_ spirals.

The four eigenvalues vary in a correlated manner along the whole set of dipeptide units. By carrying out PCA over their square roots (§[Sec sec2.2]2.2), we identified three principal components that account for 99.6% of the total variance. These define the basis of a three-dimensional space on the angstrom scale, which we denote the DipSpace (dipeptide-unit space; Figs. 2 ▸, 3 ▸ and 4 ▸) and its axes as pc1, pc2 and pc3. A variation of the data along the pc1 axis of the DipSpace correlates with the length of the first principal moment of inertia of the dipeptide unit (*r* = 0.96) and with *R*_g_ (*r* = 0.93). This suggests that the pc1 direction describes the extension of the dipeptide unit (Fig. 2 ▸ *a*). The pc2 and the pc3 axes of the DipSpace correlate weakly with the second (*r* = −0.64) and third (*r* = −0.50) axes of inertia of the dipeptide unit, respectively.

The three dimensions of the DipSpace embed the information contained in the dihedral and stretching angles. Their mapping on the Ramachandran plot is shown in Fig. 2 ▸(*b*). Similarly, the mapping of various dihedral and torsion angles on the DipSpace shows their relation to each other, as depicted in Fig. 4 ▸. We observe that a continuous walk through the DipSpace is not necessarily a continuous walk through the Ramachandran plot. Importantly, no linear correlation was identified between the DipScore and any of the three angles usually considered for the description of protein-backbone conformation (with *r* = −0.04, −0.16 and 0.08 between the computed DipScores and τ, φ and ψ, respectively).

We further illustrate the meaning of the DipSpace axes by fixing two DipSpace coordinates to a given value while varying the third one (Fig. 2 ▸ and Supplementary Videos S1, S2 and S3). The pc1 axis describing the extension of the dipeptide unit can be exemplified as a transition between a P_II_ spiral and a β-strand (Hollingsworth & Karplus, 2010 ▸; Fig. 2 ▸ *b*) or between a helical and an extended conformation (Supplementary Video S1). The pc2 direction describes the twist of the two peptide planes with respect to each other, for example a transition between a P_II_ spiral and a γ-turn (Hollingsworth & Karplus, 2010 ▸; Fig. 2 ▸ *b* and Supplementary Video S2). Finally, the pc3 axis describes the dipeptide bending, similar to a transition between a helical conformation and a δ-turn (Hollingsworth & Karplus, 2010 ▸; Fig. 2 ▸ *b* and Supplementary Video S3).

**Figure 3:**
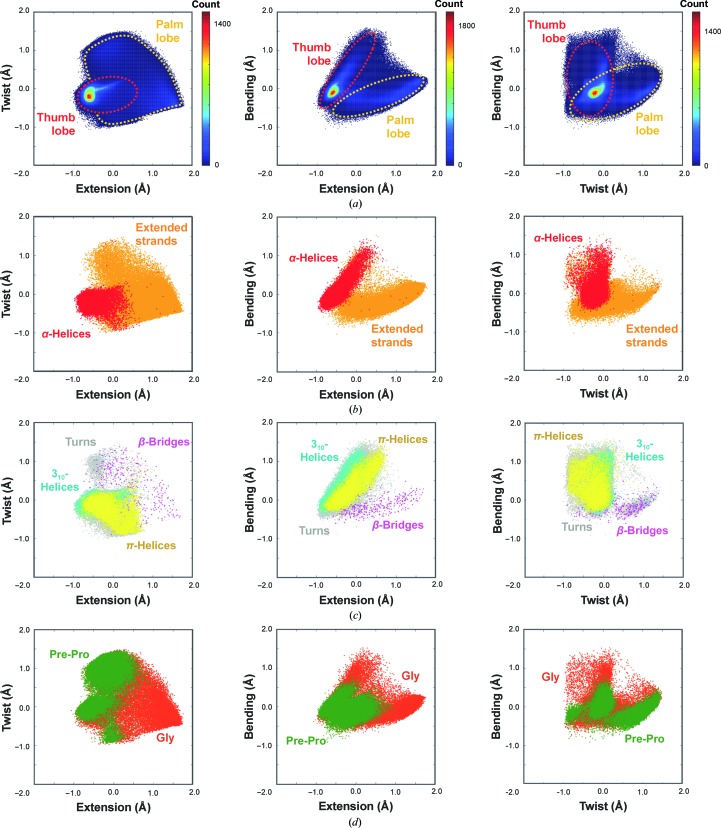
Representation of the three-dimensional DipSpace. (*a*) Joint distribution of pc_1_ (extension) and pc_2_ (twist), pc_1_ (extension) and pc_3_ (bending), and pc_2_ (twist) and pc_3_ (bending). The two main lobes are marked by dashed lines. Distribution of (*b*) α-helices and extended strands, (*c*) turns, β-bridges, π-­helices and 3_10_-helices, as annotated by *DSSP*, and (*d*) glycine and pre-proline residues (the identity corresponds to the middle C^α^ atom of the dipeptide unit).

**Figure 4:**
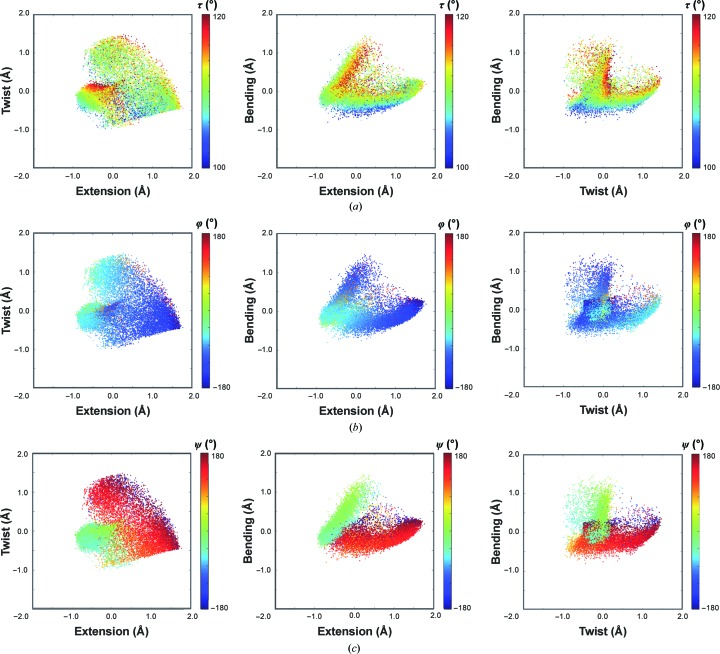
The DipSpace coloured according to (*a*) the τ stretching angle, (*b*) the Ramachandran φ dihedral angle and (*c*) the Ramachandran ψ dihedral angle.

The distribution of the conformations in the DipSpace resembles the shape of a hand, with a flatter palm, a cylindrical thumb and a thin connecting layer (Fig. 3 ▸ *a*). The thumb lobe is mainly populated by helical conformations, with variable τ and φ angles but with ψ close to zero (Fig. 4 ▸). These dipeptide units have a moderate span of twist but considerable variation in their extension and bending (Fig. 3 ▸ *b*). The separation of 3_10_-helical and π-helical conformations reflecting the change in the τ angle is shown in Figs. 3 ▸(*c*) and 4 ▸(*a*). The palm lobe is populated by turns and extended-strand conformations, with ψ close to 180° but with variable τ and φ angles (Fig. 4 ▸). The dipeptide units there have a moderate variation in their bending, but their twist and the extension vary considerably (Figs. 3 ▸ *b* and 3 ▸ *c*). Since the most abundant conformation for a protein residue is α-helical, the DipSpace is centred close to the condensed core of the thumb lobe.

Glycines are almost everywhere in the DipSpace cloud, while prolines and residues preceding prolines fall into three specific regions with predominantly lower τ angles (Figs. 3 ▸ *d* and 4 ▸).

### 3.3. Local validation of the protein model backbone   

The DipSpace highlights conformations in the PDB and indicates the frequency of their occurrence. The area in the DipSpace occupied by the uniform-noise model spans much further (Supplementary Fig. S4). The population of a given coordinate in the DipSpace represents a statistical measure of its stereochemical plausibility, which can be evaluated using the DipScore equation (1). A value of close to 1.0 indicates a well populated region of the conformations present in the PDB with little contribution from the random model; a dipeptide unit with such a score can be regarded as most likely to be in a correct conformation. Conversely, a dipeptide unit with a score close to zero would be regarded as being in a very unusual or incorrect conformation. We define a residue to be in a favoured region of DipSpace if its DipScore is above 0.24; this includes 98% of the dipeptide units collected from the PDB. The conformations of 1.8% of the points with a DipScore between 0.24 and 0.033 we denote as allowed, and further 0.15% with a DipScore between 0.033 and 0.010 are denoted as generously allowed. A residue with a DipScore below 0.010 is regarded as an outlier.

### 3.4. Overall validation of the protein model backbone   

The mean DipScore distribution for the selected set of 538 chains (§[Sec sec2.5]2.5) shows an average of 0.91 with a variance of 0.027, is negatively skewed (γ_1_ = −2.9) and is highly peaked (γ_2_ = 9; leptokurtic). The *Z*-scores for the four moments each follow a standard normal distribution but are correlated (Supplementary Table S4). By carrying out eigendecomposition of the *Z*-score variance–covariance matrix, two principal uncorrelated components, *Z*c_1_ (83.2%) and *Z*c_2_ (14.7%), with the same mean (μ = 0) but different variances [σ^2^(*Z*c_1_) > σ^2^(*Z*c_2_)] were obtained.

From the transformation matrix **R**′ equation (8), an increase in *Z*c_1_ implies an increase in the mean and the kurtosis, with a decrease in the variance and the skewness. Therefore, the component *Z*c_1_ ‘points’ in the direction of the perfect models; a model with a positive *Z*c_1_ is better than the average, while a model with a negative *Z*c_1_ represents a structure worse than the average. Thus, the overall model quality obtained from the conformity of its DipScore distribution to the expectation can be expressed using a signed χ_score_ equation (9). The models with a positive χ_score_ are better than the average, while models with a negative χ_score_ are worse.

From the cumulative distribution of the χ_score_ equation (10), one can derive that a model can be annotated as favoured (a χ_score_ percentile above 2.0; 98% of the distribution) if its χ_score_ is higher than −2.16, as allowed if the score is between −2.16 and −2.97 (percentile between 2.0 and 0.2) and as generously allowed if the score is between −2.97 and −3.38 (percentile between 0.2 and 0.05); otherwise it is an outlier.

### 3.5. Application to the validation of deposited protein models   

**Figure 5:**
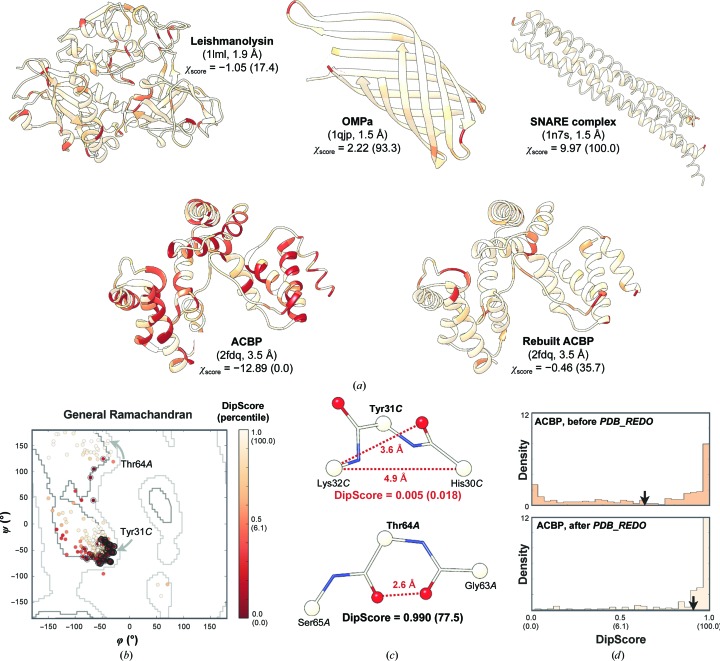
Local and overall protein model validation using the DipSpace. Values in parentheses indicate the corresponding percentiles. (*a*) Cartoon representation of the test cases, coloured by their local DipScore. The PDB codes and resolutions of the models are indicated. (*b*) General (nonglycine/nonproline) Ramachandran plot for the ACBP model. The allowed (grey) and favoured (dark grey) boundaries according to Lovell *et al.* (2003 ▸) are marked. Outliers (DipScore < 0.010; percentile < 0.05) are surrounded by a black circle and those in allowed and generously allowed regions (DipScore between 0.010 and 0.240; percentile between 0.05 and 2.0) by a light grey circle. (*c*) Ball-and-stick representation of ACBP Tyr31*C* and Thr64*A* dipeptide units, highlighting their DipScore and problematic distances. (*d*) DipScore histograms for the ACBP models. Arrows mark the average DipScore for the model.

Examples representing different scenarios in protein structural analysis and demonstrating the applicability of the DipSpace, DipScore and χ_score_ for the local and overall validation of protein models are described below (Fig. 5 ▸ *a* and Supplementary Table S5).

**Example 1**. The armadillo acyl-CoA-binding protein (ACBP; Costabel *et al.*, 2006 ▸; PDB entry 2fdq) is an all-α protein complex refined at 3.5 Å resolution. It has a *WHAT_CHECK* Ramachandran *Z*-score (Hooft *et al.*, 1997 ▸) of −6.69 and 12 Ramachandran outliers out of 225 nonglycine/nonproline residues (Supplementary Table S5). The DipSpace indicates 13 outliers, but not all are the same (Supplementary Table S6). There are residues that are in the allowed region of the Ramachandran plot but in the disallowed area of the DipSpace, and *vice versa*. For example, Tyr31*C* located in the favoured region of the Ramachandran plot has a τ angle of 106.8° and is an outlier in the DipSpace owing to too short variable distances (C^α^_*i*−1_—C^α^_*i*+1_ of 4.9 Å and O_*i*_—C^α^_*i*+1_ of 3.6 Å; Fig. 5 ▸ *c* and Supplementary Fig. S2). Interestingly, this residue is not marked as problematic in the PDB validation report. Another example is Thr64*A* (Fig. 5 ▸ *c*), in which the dipeptide interatomic distances fall in the peaks of their distributions, except for O_*i*−1_—O_*i*_ (2.6 Å), thus pulling this residue into the favoured region of the DipScore. In the Ramachandran plot this residue is near the border of the allowed region (Fig. 5 ▸ *b*).

A considerable improvement in the ACBP model geometry was obtained using *PDB_REDO* (Fig. 5 ▸ *a* and Supplementary Table S5). The short O_*i*−1_—O_*i*_ distance around Thr63*A* increased by about 1.0 Å without any distortion of the other distances. The Tyr31*C* τ angle increased to 110.5°, with a concurrent increase of the C^α^_*i*−1_—C^α^_*i*+1_ and O_*i*_—C^α^_*i*+1_ distances. The improvement in the ACBP backbone geometry is also demonstrated by an increase of its χ_score_ to −0.46 and in the percentile to 36 (Figs. 4 ▸ *d* and 5 ▸ *a* and Supplementary Table S5).

**Examples 2, 3 and 4**. These models represent all-β, coiled-coil and mixed structures without conformational deficiencies. All have a χ_score_ within the expected range (Fig. 5 ▸ *a* and Supplementary Table S5). We notice that the value of χ_score_ for protein models without problematic regions may be affected by the protein secondary-structure content. For example, a fully helical geometrically perfect model may have most of its C^α^ atoms in the condensed core of the DipSpace thumb lobe, which has a DipScore close to 1.0. On the contrary, C^α^ atoms in an all-β model without geometrical problems have a broader area of allowed coordinates in the DipSpace. Therefore, the DipScore distribution of an all-α model has different characteristics from those of an all-β model and mixed α–β models (Supplementary Fig. S5).

### 3.6. Application to the detection of strained residues with potential functional relevance   

For the set of dipeptide units collected from the PDB, a main-chain environment for a residue is defined as allowed if its DipScore is above 0.24; this includes 98% of the residues in the PDB-derived data set. A low DipScore value is statistically also allowed, but it may indicate an incorrect geometry. At the same time, it may also indicate an unusual geometry owing to other reasons, as demonstrated below.

**Figure 6:**
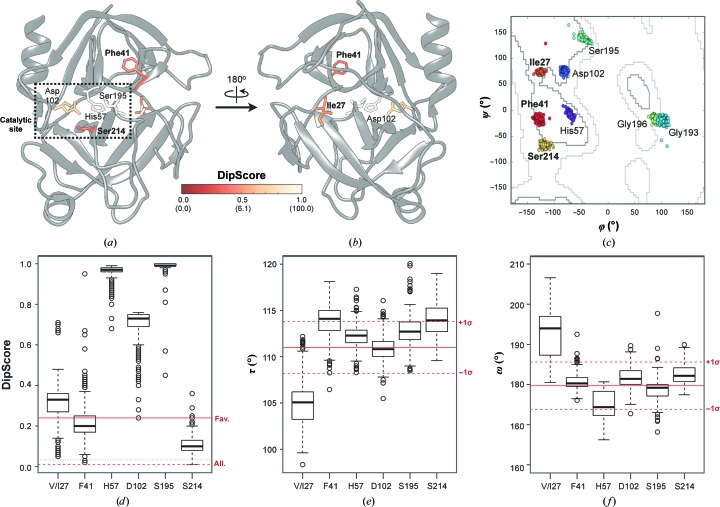
DipSpace-based analysis of the trypsin family. (*a*, *b*) Cartoon representation of the porcine trypsin model (PDB entry 2a31) viewed from two perspectives. The catalytic residues as well as Phe41 and Ile27 are shown in stick representation and are coloured by DipScore. Values in parentheses indicate the corresponding DipScore percentiles. (*c*) Ramachandran plot for the corresponding catalytic residues as well as Phe41 and Ile27 in all 350 trypsin models considered. The allowed (grey) and favoured (dark grey) boundaries according to Lovell *et al.* (2003 ▸) are marked. (*d*, *e*, *f*) Box plots for the (*d*) DipScore, (*e*) τ angle and (*f*) ω angle for the four main catalytic residues as well as Phe41 and (Val)Ile27. (*d*) The favoured, allowed and generously allowed DipScore thresholds are marked by straight, dotted and dashed red lines, respectively. (*e*, *f*) The expected average and the 1σ intervals according to MacArthur & Thornton (1996 ▸) and Engh & Huber (2006 ▸) are marked by straight and dashed red lines, respectively.

In the trypsin serine protease structures, the residues His57, Asp102, Gly193, Ser195, Gly196 and Ser214 are annotated as catalytic [residue numbering corresponds to the reference porcine model (PDB entry 2a31; Transue *et al.*, 2006 ▸)]. His57, Asp102 and Ser195 form the catalytic triad, Gly193 builds the oxyanion hole with Ser195, and Gly196 stabilizes the intermediate state. Ser214 is highly conserved in serine proteases and has been proposed for inclusion in a catalytic tetrad (Meyer *et al.*, 1988 ▸). This residue assists in delocalization of the charge of His57, forms contacts with the substrate and the other catalytic residues (Meyer *et al.*, 1988 ▸; Corey *et al.*, 1992 ▸; Peisach *et al.*, 1999 ▸; Krem *et al.*, 2002 ▸; Fuhrmann *et al.*, 2004 ▸), and is located in a cleft between the two structural domains (Figs. 6 ▸ *a* and 6 ▸ *b*; Kraut, 1977 ▸; Meyer *et al.*, 1988 ▸).

While all residues annotated as catalytic fall within allowed or favoured regions of the Ramachandran plot (Fig. 6 ▸ *c*), Ser214 has systematically the lowest DipScore among the structures of the trypsin family (0.11 ± 0.05; Figs. 6 ▸ *a*, 6 ▸ *b* and 6 ▸ *d*). From the average DipScore distribution, we obtain that only 0.8 residues out of 100, on average, have a DipScore of this value or lower. This low DipScore indicates an unusual, but still statistically plausible, main-chain conformation, which may well occur in an overall good-quality model. However, it is extremely unlikely that the same residue has such a low DipScore in all 350 models ‘by chance’. The strain in the geometrical environment for Ser214 is not seen in its φ/ψ angles, but the long O_*i*_—O_*i*−1_ distance of 6.1 ± 0.1 Å, which is about 1.0 Å longer than is typically observed in the PDB (Supplementary Fig. S2*b*), together with a wide τ angle (Fig. 6 ▸ *e*), are definitely unusual. This may be explained by its catalytic role and interaction with the neighbouring side chains.

In addition, in all 350 trypsin models residues 27 and 41 showed consistently low average DipScore values (Fig. 6 ▸ *d*): 0.33 ± 0.10 and 0.22 ± 0.10 with percentiles 3.1 and 1.8, respectively. In 89.9% of the models there is a valine at position 27. In the reference model an isoleucine is present at this position. In 98.3% of the cases it precedes a *trans* proline. All residues at position 27 populate favoured regions of the Ramachandran plot (Fig. 6 ▸ *c*). The lower DipScore for position 27 is a result of a long O_*i*_—O_*i*−1_ distance of 5.4 ± 0.1 Å, an unusually small τ angle (2.2 ± 0.8 standard deviations lower than the mean value; Fig. 6 ▸ *e*; Engh & Huber, 2006 ▸; Berkholz *et al.*, 2009 ▸) and a deviation from the peptide plane between residues 27 and 28 (the ω angle is 2.1 ± 1.2 standard deviations larger than the average for the *trans* peptide; Figs. 6 ▸ *f* and 7 ▸ *c*). This residue is located in a loop on the surface of the protein, far from the catalytic site, at the start of the first β-barrel domain (Fig. 6 ▸ *b*).

**Figure 7:**
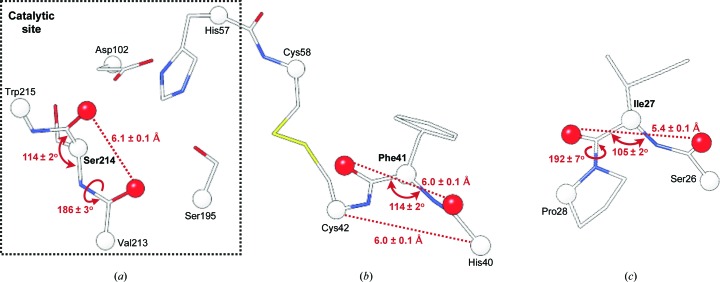
The geometrical characteristics of strained residues in trypsin. (*a*) Ser214 in the catalytic site, (*b*) Phe41 close to the active site and (*c*) Ile27 far from the catalytic site.

Position 41 is located close to the catalytic pocket (Figs. 6 ▸ *a* and 7 ▸ *b*) and is known to interact with trypsin inhibitors (Jaśkiewicz *et al.*, 1998 ▸; Batt *et al.*, 2015 ▸; Cui *et al.*, 2015 ▸). In 98.2% of the cases it is a phenylalanine. Similar to Val/Ile27, Phe41 is in the allowed region of the Ramachandran plot (Fig. 6 ▸ *c*). Although it has a helical C^α^_*i*−1_–C^α^_*i*+1_ distance of 6.0 ± 0.1 Å, its other variable distances are close to the upper limit of the stranded conformation (Fig. 7 ▸ *b*), which results in a wider τ angle (Fig. 6 ▸ *e*). Such geometry allows the Phe41 carbonyl O atom involved in interaction with the inhibitor to face the binding pocket and is possibly stabilized by a Cys42–Cys58 disulfide bridge (Fig. 7 ▸ *b*).

The conserved geometrical distortions of Ser214, Val/Ile27 and Phe41 are supported by the experimental electron density from the EDS (Kleywegt *et al.*, 2004 ▸), with an RSCC higher than 0.98 for the reference porcine structure.

Additionally, we found that refined models with identical sequences and reasonable PDB validation reports and that are superimposable with a main-chain r.m.s.d. of 0.14 Å may have very different values of χ_score_. For example, the bovine trypsin model PDB entry 1g36 determined at 1.9 Å resolution has a χ_score_ percentile of 77.0, while PDB entry 1o2q at 1.5 Å resolution has a percentile of 2.5. Although both represent the same molecule, many of the ‘fixed distances’ and ω angles for the 1o2q model vary too greatly from their typical values. Running *PDB_REDO* on the 1o2q model and the experimental data from the isomorphous PDB entry 2fx6 (no experimental data are available for 1o2q) resulted in a χ_score_ percentile of 30.6.

## 4. Conclusions   

Distance geometry has been extensively used in structural biology, from NMR structure determination (Crippen & Havel, 1988 ▸) to protein structure prediction (Kloczkowski *et al.*, 2009 ▸) and comparison (Schneider, 2000 ▸). It has also been applied to the conformational description of small molecules (Dixon, 2010 ▸) and has proved to be powerful for the identification of ligands in electron-density maps (Carolan & Lamzin, 2014 ▸). Our results demonstrate that it can also be efficiently used for the description of protein backbone conformation and the validation of protein models.

In summary, the method evaluates a C^α^ position in its dipeptide-unit environment, described as a matrix of the interatomic distances. The first eigendecomposition for the whole PDB-derived data converts the distances to the orthogonal eigenvalues. The second eigendecomposition eliminates the interdependence of these eigenvalues as they change in a related way throughout the PDB. This embeds geometrical information about the backbone atoms around each C^α^ atom in a protein model within a unified orthogonal Euclidean three-dimensional space where the three axes are on the same absolute scale.

The DipSpace axes do not correlate to any of the Ramachandran angles or to the τ stretching angle; instead, they represent a relative extension, twist and bending of the two peptide planes within the dipeptide unit. Thus, a point in the DipSpace is a summary of the interatomic distances around a given C^α^ atom. We note that the location of the central C^α^ atom in a dipeptide unit is particularly important as it may highlight the distortions of the ‘fixed distances’ and discriminate between *trans* and *cis* peptides. The higher dimensionality of the DipSpace makes it intrinsically more informative compared with other two-dimensional or one-dimensional geometry descriptors, but a joint use of all available geo­metrical information is certainly the most advantageous.

The DipSpace, reflecting the information that is present in the PDB, along with the addition of the noise model, allows the computation of a DipScore for each individual residue and provides a local evaluation of protein backbone conformation. We propose that a residue and its environment may require additional inspection if it has a DipScore percentile around 2.0 or lower, particularly when its stretched main chain is evaluated as a DipScore outlier. Any outlier should be considered appropriately during structure determination or analysis, as it may indicate something incorrect in our understanding, or may point to something new and interesting. A low DipScore value in refined protein models may sometimes reflect a stretched main-chain stereochemistry for reasons of natural functional importance, if this is supported by other experimental evidence, for example its structural conservation in a protein family and/or its fit to the electron density. As one example, we have presented three such residues in the structures of trypsin with systematically low DipScores but allowed Ramachandran angles. The availability of experimental data supporting these residues having an unusual backbone conformation for reasons of their likely functional or structural relevance may be of interest for further research.

The distribution of the individual DipScores within a given protein model can be compared with that of the deposited protein models. This is performed through the third eigen­decomposition (of the moments of DipScore distributions in the selected protein structures) and results in the overall χ_score_. This provides a measure of the agreement of the overall protein model with the observed overall distributions of conformations and geometries for the models deposited in the PDB, and can be regarded as resembling the concept of the *WHAT_CHECK* Ramachandran *Z*-score. In our case, the χ_score_ follows a χ distribution where a sign is included to separate the protein models that are better or worse than the average model deposited in the PDB. It can therefore be used for the detection of protein models with regions of unusual conformations or geometry of *trans* peptide units. One would generally expect models with a poor Ramachandran plot or *WHAT_CHECK* *Z*-score to also display a poor DipSpace χ_score_, but variations can be observed, as shown by the examples in Supplementary Table S5. Similarly to the local validation of protein backbone, we propose that additional inspection or refinement may be undertaken for a model with a χ_score_ that is too low, as we demonstrate by the bovine trypsin and armadillo acyl-CoA-binding protein examples. We note that the χ_score_ is not very sensitive to random coordinate errors, although purely random errors rarely occur in structure determination. However, even a random additional coordinate error of 0.1 Å should cause the χ_score_ percentile to become zero, indicating that the model is geometrically an outlier.

The presented way to compute the DipScore does not differentiate the identity of the residue, as we have yet to identify specific residue-preferred areas in the DipSpace, other than the prolines and pre-prolines mentioned above. It will certainly be of interest to further investigate the DipScore distributions for other residues and *cis*-prolines. Another direction to pursue could be the addition of weights or a deliberate narrowing of the distributions of the intra-dipeptide distances, so that the DipSpace becomes tuned to a particular geometrical feature, for example the O_*i*−1_—O_*i*_ distance. The use of other deliberately biased random-‘noise’ models could also adjust the method towards different approaches for model building or validation.

The developed method, which is implemented as the *DipCheck* software, is available as a web service from http://cluster.embl-hamburg.de/dipcheck.

## Supplementary Material

Supplementary Figures and Tables.. DOI: 10.1107/S2052252517008466/jt5019sup1.pdf

Legends for the Supplementary Videos.. DOI: 10.1107/S2052252517008466/jt5019sup2.pdf

Supplementary Video S1.. DOI: 10.1107/S2052252517008466/jt5019sup3.mov

Supplementary Video S2.. DOI: 10.1107/S2052252517008466/jt5019sup4.mov

Supplementary Video S3.. DOI: 10.1107/S2052252517008466/jt5019sup5.mov

DipSpace grid data; values of the DipScores in the DipSpace. DOI: 10.1107/S2052252517008466/jt5019sup6.xls

## Appendix A Transformation to the DipSpace

For a given dipeptide unit, its coordinates (*P*) in the DipSpace can be obtained from

where **L** is the column vector of the square roots of the four eigenvalues for the given dipeptide unit,

 is the column vector of their means among all selected dipeptides,

and **R** is the transformation matrix obtained by PCA,

## Appendix B Calculation of the χ_score_

The two decorrelated *Z*-scores (*Z*c_*i*_) can be calculated with

where **Z** is the vector of the four *Z*-scores (*Z_i_*) for the given model, **Z** = (*Z*_1_, *Z*_2_, *Z*_3_, *Z*_4_), and **R**′ is the transformation matrix,

Over the set of 516 chains, *Z*c_1_ and *Z*c_2_ have a mean value of zero but different variances [σ^2^(*Z*c_1_) = 3.322 and σ^2^(*Z*c_2_) = 0.585]. This allows their combination,

to follow a χ distribution with [σ^2^(*Z*c_1_) + σ^2^(*Z*c_2_)]/σ^2^(*Z*c_1_) = 1.176 degrees of freedom.

By multiplying this by the sign of the highest uncorrelated component *Z*c_1_, we define a signed χ_score_ characterizing the overall deviation of the DipScore distribution for the model in question from those for the set of good models,
