# Supplementary material for: A distance geometry-based description and validation of protein main-chain conformation
Source: IUCrJ. 2017 Aug 8;4(Pt 5):657–70. doi: 10.1107/S2052252517008466 (PMC5619857; doi:10.1107/S2052252517008466)
Supplement: Supplementary file 1 [file m-04-00657-sup1.pdf]

IUCrJ

Volume 4 (2017)

Supporting information for article:

A distance geometry-based description and validation of protein main-chain conformation

Joana Pereira and Victor S. Lamzin

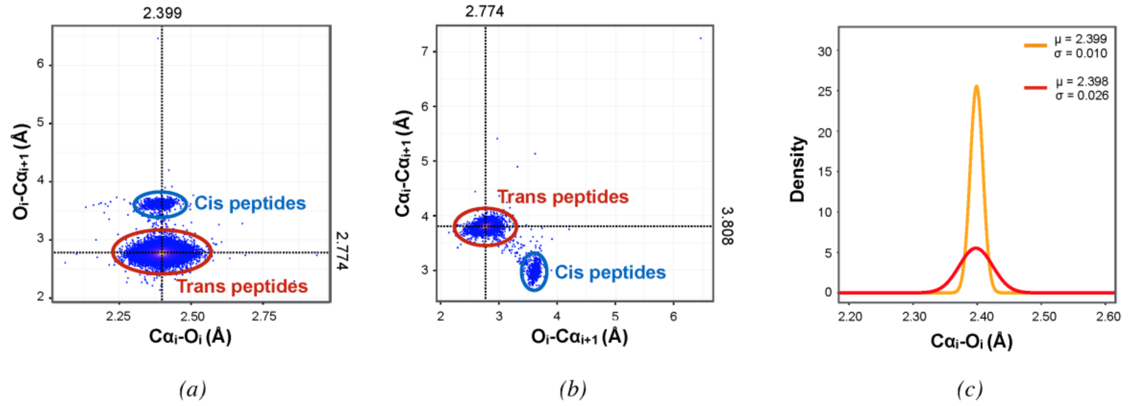

Figure S1 Distribution of the fixed distances in peptide units. (*a-b*) Joint distribution of $C\alpha_i$-O_i and $C\alpha_i$-$C\alpha_{i+1}$ distances and O_i-$C\alpha_{i+1}$ and $C\alpha_i$-$C\alpha_{i+1}$ distances. Dashed lines mark the median value of each distribution. (*c*) Normal mixed-model description of the $C\alpha_i$-O_i distance distribution. Continuous lines show the Gaussian functions fitting the data: the major component with the mixing proportion of about 0.7 (in orange), and the minor component with the proportion of about 0.3 (in red).

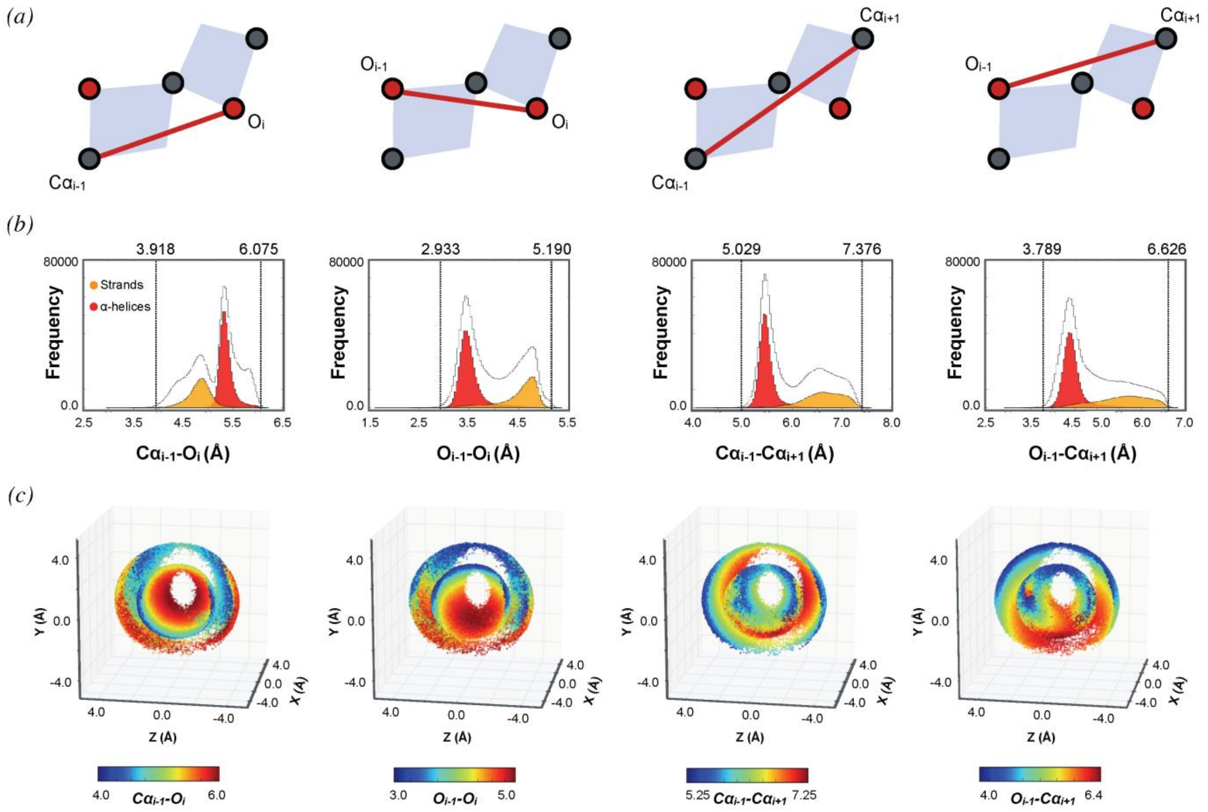

Figure S2 The variable distances. (*a*) The dipeptide unit atoms involved. (*b*) Histograms showing their one-dimensional distributions after filtering by the fixed distances, as described in the text. The distributions are multimodal; the helical regions are in red and the stranded regions in orange. Vertical dashed lines in each distribution mark the boundaries of the interval containing 99.8% of the points. (*c*) The mapping of the variable distances on the three-dimensional real space occupied by each atom

in the dipeptide unit when they were aligned by their first peptide plane so that each Cα_{i-1} was on the positive side of x axis, Cα_i at the origin and O_{i-1} in the first quadrant of the xy plane.

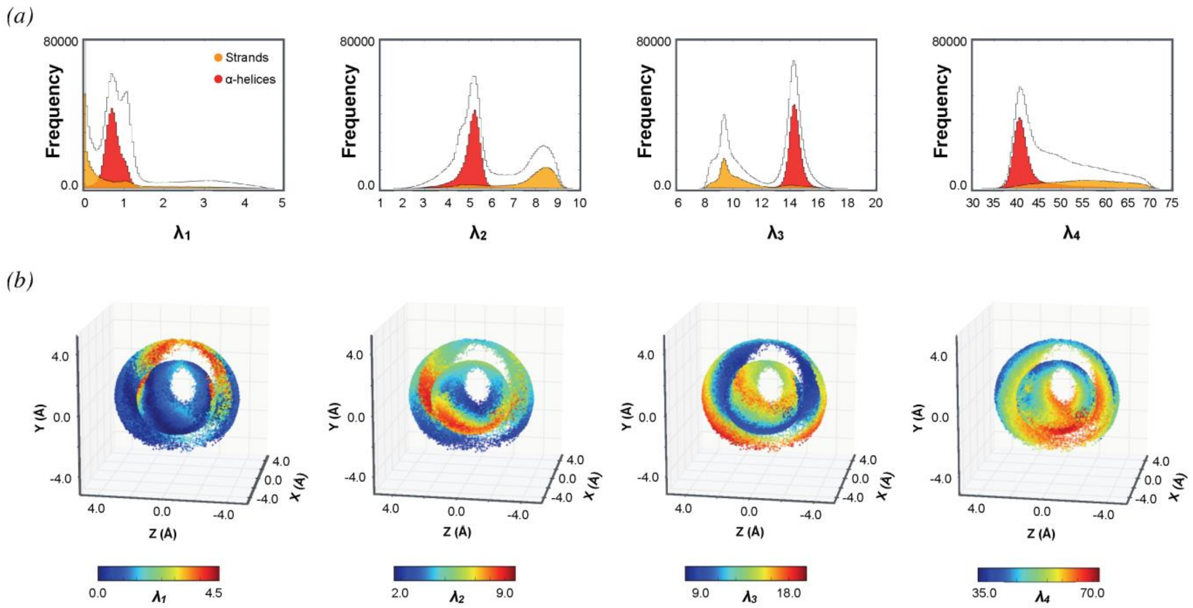

Figure S3 The four eigenvalues of the distance-squared matrix. (*a*) Histograms showing their distributions and depicting the contribution of the structural preferences of the main-chain (helices and strands). (*b*) The mapping of the eigenvalues on the three-dimensional real space occupied by each atom in the dipeptide unit when they were aligned by their first peptide plane so that each Cα_{i-1} was on the positive side of x axis, Cα_i at the origin and O_{i-1} in the first quadrant of the xy plane.

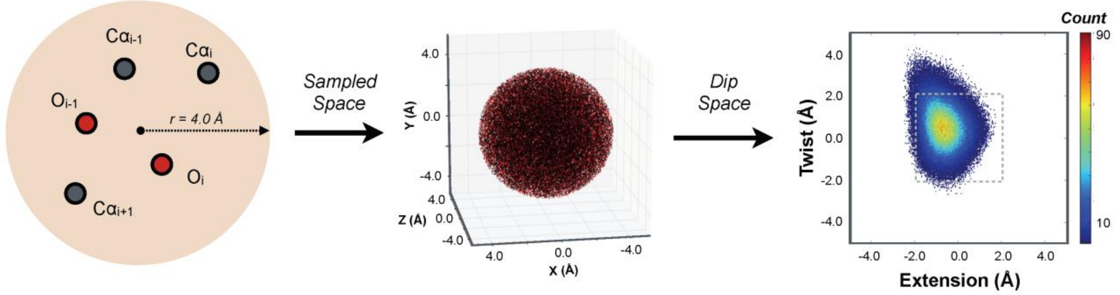

Figure S4 Modelling of the noise as a random sampling of 5 atoms inside a sphere of 4.0 Å radius. Their sampled space and the projection on the first two axes of the DipSpace are shown. Grey dashed lines mark the boundaries of the location of dipeptides in the DipSpace.

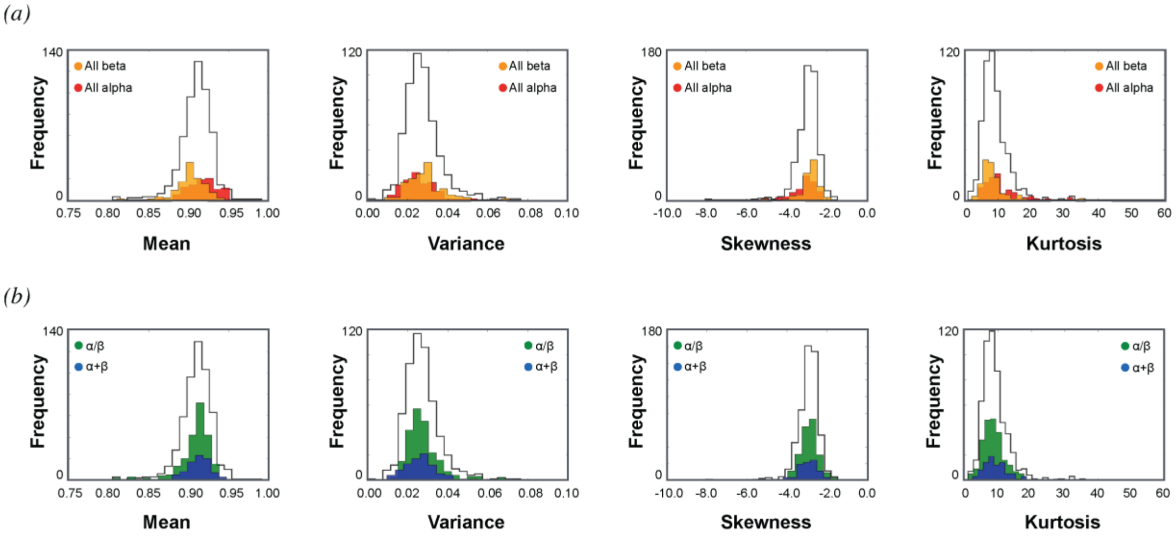

Figure S5 Distributions of the four central moments of DipScore distributions calculated for the set of 538 protein chains. The contribution of the different fold classes is depicted for (*a*) all-alpha and all-beta, (*b*) mixed alpha and beta structures.

Table S1 Parameters of the Gaussian functions describing the distribution of the three fixed distances in trans peptide units. μ, the mean (in Å); σ, the standard deviation; ν, the mixing proportion.

	$C\alpha_i$-O_i	$C\alpha_i$- $C\alpha_{i+1}$	O_i-$C\alpha_{i+1}$
Major Gaussian Function			
μ	2.399	3.808	2.774
σ	0.010	0.017	0.033
ν	0.64	0.63	0.77
Minor Gaussian Function			
μ	2.398	3.812	2.775
σ	0.026	0.034	0.062
ν	0.36	0.36	0.23

Table S2 Target values for the calculation of the Z-scores for the first four central moments of the DipScore distribution.

	Average (m_1)	Variance (m_2)	Skewness (m_3)	Kurtosis (m_4)
μ	0.9002	0.02686	-2.916	8.998
σ	0.0156	0.00717	0.410	3.128

Table S3 Linear correlation coefficients (r) between the square-roots of the absolute values of the four negative eigenvalues of distance-squared matrices and the variable distances (in Å scale) as well as between the absolute values of the four negative eigenvalues of distance-squared matrices and the three principal components of the *xyz* variance-covariance matrices (Dc_i) of 5-atom dipeptide units (in $Å^2$ scale).

	$\sqrt{\lambda_1}$	$\sqrt{\lambda_2}$	$\sqrt{\lambda_3}$	$\sqrt{\lambda_4}$
Variable distances				
$C\alpha_{i-1}$-O_i	-0.297	0.864	-0.666	-0.259
O_{i-1}-O_i	0.808	-0.297	0.420	-0.058
$C\alpha_{i-1}$-$C\alpha_{i+1}$	0.889	-0.905	0.705	-0.077
O_{i-1}-$C\alpha_{i+1}$	0.958	-0.580	0.528	-0.423
	λ_1	λ_2	λ_3	λ_4
Dipeptide unit principal components (Dc_i)				
Dc_1	1.000	-0.666	0.573	-0.161
Dc_2	-0.586	0.941	-0.657	-0.434
Dc_3	-0.705	0.216	-0.085	0.188
R_g^2	0.980	-0.533	0.525	-0.304

Table S4 Correlation matrix of the four Z-scores (Z_i) calculated for the four first central moments of DipScore distributions for the 516 selected protein chains.

	**Average (Z_1)**	**Variance (Z_2)**	**Skewness (Z_3)**	**Kurtosis (Z_4)**
**Average (Z_1)**	1	-0.889	-0.803	0.776
**Variance (Z_2)**	-	1	0.584	-0.609
**Skewness (Z_3)**	-	-	1	-0.983
**Kurtosis (Z_4)**	-	-	-	1

Table S5 Main chain quality indicators for the five test cases (glycines and prolines are excluded). Dipeptide units that fall in allowed areas of the general Ramachandran plot, as defined by Lovell *et al.*, are referred as allowed, and those that fall outside the allowed and favoured regions referred as outliers. Dipeptide units with a DipScore below 0.01 are referred as outliers and those with a score within 0.01 and 0.24 as allowed (combining allowed and generously allowed). Their percentage is shown in parentheses. The model overall χ_{score}, χ_{score} percentile and Ramachandran Z-score, as calculated by WHAT_CHECK, are also presented.

	**1LML** Alpha-beta	**1QJP** Purely Beta	**2FDQ** Before PDB_REDO	**2FDQ** After PDB_REDO	**1N7S** Purely helical
Ramachandran (% is shown in parentheses)					
Outliers	0 (0.0)	0 (0.0)	12 (5.2)	0 (0.0)	1 (0.4)
Allowed	8 (2.1)	0 (0.0)	62 (26.8)	12 (5.3)	2 (0.8)
DipSpace (DipScore) (% is shown in parentheses)					
Outliers	0 (0.0)	0 (0.0)	13 (5.6)	0 (0.0)	0 (0.0)
Allowed	5 (1.3)	1 (1.0)	41 (17.7)	7 (3.1)	1 (0.4)
Model C_{score} (overall DipScore distribution)					
C_{score}	-1.05	2.22	-12.89	-0.46	9.97
Percentile	17.4	98.3	0.0	35.7	100.0
Ramachandran Z-score					
Z-score	-1.43	-0.301	-6.69	-0.54	3.99

Table S6 Non proline/glycine Ramachandran-plot or DipSpace outliers for the crystallographic model of the armadillo acyl-CoA-binding protein (ACBP) (PDB ID 2fdq), out of a total of 231 residues evaluated.

Residue	φ, ψ, τ (°)	Ramachandran status	DipScore	DipSpace status
Ala9A	-56.3, -75.7, 105.1	Outlier	0.036	Allowed
Glu10A	-39.3, -47.2, 110.0	Allowed	0.004	Outlier
Val12A	-37.2, -39.6, 113.7	Allowed	0.000	Outlier
Lys16A	-37.9, -29.4, 118.7	Outlier	0.773	Favoured
Asp22A	-35.1, -40.6, 111.3	Allowed	0.000	Outlier
Ile39A	-33.8, 137.6, 115.3	Outlier	0.990	Favoured
Thr64A	-40.3, 153.5, 113.1	Outlier	0.990	Favoured
Ala20B	-39.2, 157.9, 111.1	Outlier	0.984	Favoured
Asp22B	-53.3, -75.8, 104.8	Outlier	0.001	Outlier
Glu23B	-32.1, -55.0, 111.3	Allowed	0.001	Outlier
Phe26B	-32.1, -54.1, 109.7	Allowed	0.001	Outlier
Asp48B	-66.3, 44.7, 108.6	Outlier	0.029	Gen. allowed
Lys66B	-29.0, -54.9, 106.7	Allowed	0.000	Outlier
Tyr73B	-47.7, -95.5, 108.7	Outlier	0.407	Favoured
Ile74B	-28.0, -43.4, 110.5	Outlier	0.000	Outlier
Ile27C	-57.2, -74.8, 109.1	Outlier	0.121	Allowed
Tyr28C	-34.3, -72.8, 111.7	Outlier	0.005	Outlier
Tyr31C	-51.3, -29.8, 106.8	Favoured	0.005	Outlier
Gln33C	-47.9, 2.1, 113.6	Outlier	0.857	Favoured
Lys52C	-44.4, -34.8, 109.7	Favoured	0.009	Outlier
Gln60C	-45.2, -31.1, 110.6	Allowed	0.004	Outlier
Asp75C	-36.0, -71.8, 107.3	Allowed	0.000	Outlier

Table S7 Summary of trypsin models used for the detection of conserved strained residues.

PDBID	Resolution (Å)	Number of residues	χ_{Score}	χ_{Score} percentile
1ANC	2.2	226	-0.87	22.4
1AND	2.3	226	-0.87	22.2
1AUJ	2.1	226	0.68	71.9
1AZ8	1.8	226	-0.33	40.1
1BIT	1.83	240	-0.19	44.7
1BJU	1.8	226	-1.22	13.4
1BJV	1.8	226	-0.92	21.0
1BRA	2.2	226	-0.96	19.9
1BTP	2.2	232	-1.82	4.4
1BTY	1.5	232	-0.98	19.2
1BTZ	2	232	-1.83	4.2
1C1N	1.4	226	-1.1	16.1
1C1O	1.4	226	-1.49	8.4
1C1P	1.37	226	-0.69	28.0
1C1Q	1.37	226	-0.46	35.6
1C1R	1.37	226	-0.86	22.6
1C1S	1.63	226	-1.82	4.3
1C1T	1.37	226	-0.71	27.3
1C2E	1.65	226	-1.07	16.8
1C2F	1.7	226	-1.34	10.9
1C2G	1.65	226	-0.75	25.8
1C2H	1.4	226	-1.17	14.5
1C2I	1.47	226	-1.34	10.9
1C2J	1.4	226	-1.26	12.6
1C2K	1.65	226	-1.17	14.5
1C2L	1.5	226	-1.68	5.8
1C2M	1.4	226	-1.2	13.8

Table S7 Summary of trypsin models used for the detection of conserved strained residues (cont.).

PDBID	Resolution (Å)	Number of residues	χ_{Score}	χ_{Score} percentile
1C5P	1.43	226	-1.09	16.4
1C5Q	1.43	226	-1.22	13.3
1C5R	1.47	226	-1.38	10.2
1C5S	1.36	226	-1.02	18.2
1C5T	1.37	226	-0.92	20.8
1C5U	1.37	226	-0.31	40.7
1C5V	1.48	226	-1.43	9.4
1CE5	1.9	226	0.42	63.0
1DPO	1.59	226	-0.2	44.4
1EB2	2	226	-1.08	16.6
1F0T	1.8	247	0.91	78.8
1F0U	1.9	247	-0.53	33.2
1FMG	1.9	226	-0.22	43.8
1FN6	1.8	226	0.49	65.5
1FNI	1.6	226	-0.3	41.0
1FXY	2.15	231	0.8	75.5
1G36	1.9	226	0.85	77.0
1G3B	1.8	231	0.46	64.3
1G3C	1.8	231	0.81	75.8
1G3D	1.8	231	0.49	65.3
1G3E	1.8	231	0.9	78.7
1GHZ	1.39	226	-0.49	34.5
1GI0	1.42	226	-0.66	28.9
1GI1	1.42	226	-0.79	24.7
1GI2	1.38	226	-1.34	11.0
1GI4	1.37	226	-1.35	10.8
1GJ6	1.5	226	-1.63	6.5

Table S7 Summary of trypsin models used for the detection of conserved strained residues (cont.).

PDBID	Resolution (Å)	Number of residues	χ_{Score}	χ_{Score} percentile
1H4W	1.7	227	1.89	96.2
1HJ8	1	225	2.4	98.9
1J14	2.4	226	0.22	56.3
1J15	2	226	0.66	71.1
1J16	1.6	226	0.65	70.8
1J17	2	226	0.66	71.3
1J8A	1.21	226	0.41	62.6
1JIR	2	226	0.13	53.4
1K1I	2.2	226	0.23	56.5
1K1J	2.2	226	0.47	64.8
1K1L	2.5	226	-0.57	31.7
1K1M	2.2	226	0.72	73.0
1K1N	2	226	0.04	50.8
1K1O	2	226	0.5	65.6
1K1P	1.9	226	0.7	72.4
1LQE	2.2	247	0.68	71.7
1MAY	1.8	226	-1.46	8.9
1MBQ	1.8	223	0.48	65.0
1MTS	1.9	226	0.88	78.0
1MTU	1.9	226	0.88	78.0
1MTV	1.9	226	0.84	76.8
1MTW	1.9	226	0.91	78.9
1N6X	1.4	226	0.37	61.4
1N6Y	1.4	226	0.22	56.3
1NC6	1.9	226	0.97	80.4
1O2L	1.68	226	-1.22	13.3
1O2M	1.69	226	-1.89	3.7

Table S7 Summary of trypsin models used for the detection of conserved strained residues (cont.).

PDBID	Resolution (Å)	Number of residues	χ_{Score}	χ_{Score} percentile
1O2N	1.5	226	-1.41	9.7
1O2Q	1.5	226	-2.07	2.5
1O2T	1.62	226	-1.5	8.2
1O2U	1.41	226	-0.89	21.8
1O2V	1.5	226	-1.75	5.0
1O2W	1.38	226	-1.14	15.3
1O2X	1.46	226	-1.15	15.0
1O2Y	1.45	226	-1.02	18.1
1O30	1.55	226	-1.41	9.7
1O36	1.7	226	-1.81	4.4
1O37	1.45	226	-0.98	19.2
1O38	1.38	226	-1.06	17.2
1O39	1.59	226	-1.42	9.6
1O3B	1.75	226	-1.64	6.3
1O3C	1.64	226	-1.95	3.2
1O3D	1.33	226	-1.16	14.6
1O3E	1.64	226	-1.66	6.1
1O3F	1.55	226	-1.72	5.4
1O3G	1.55	226	-1.72	5.4
1O3L	1.4	226	-1.12	15.6
1OS8	1.55	226	0.12	53.2
1OSS	1.93	226	0.05	51.2
1OYQ	1.9	226	0.84	76.8
1PPC	1.8	226	-0.54	32.8
1PPH	1.9	226	-1.42	9.5
1PPZ	1.23	227	0.72	73.1
1PQ5	0.85	227	1.53	92.2

Table S7 Summary of trypsin models used for the detection of conserved strained residues (cont.).

PDBID	Resolution (Å)	Number of residues	χ_{Score}	χ_{Score} percentile
1PQ7	0.8	227	0.97	80.5
1PQA	1.23	227	-1.13	15.3
1QA0	1.8	226	0.49	65.5
1QB1	1.8	226	0.16	54.2
1QB6	1.8	226	-0.1	47.4
1QB9	1.8	226	0.46	64.3
1QBN	1.8	226	0.66	71.3
1QBO	1.8	226	0.34	60.2
1QCP	1.8	226	0.39	61.9
1QL7	2.1	226	-0.28	41.8
1QL8	3	226	-1.29	11.8
1QL9	2.3	226	0.44	63.7
1QQU	1.63	226	0.49	65.3
1RXP	1.7	226	0.6	69.2
1S0Q	1.02	226	-0.35	39.5
1S0R	1.02	226	-0.74	26.3
1S5S	1.4	226	-0.51	33.7
1S6F	1.8	226	-0.44	36.2
1S6H	1.45	226	0.69	72.0
1S81	1.7	226	-1	18.6
1S82	1.85	226	-0.48	34.8
1S83	1.25	226	0.46	64.2
1S84	1.85	226	-0.76	25.6
1S85	2.2	226	-1.1	16.2
1SGT	1.7	226	-0.51	33.9
1TGC	1.8	232	0.22	56.2
1TGT	1.7	232	-0.69	27.9

Table S7 Summary of trypsin models used for the detection of conserved strained residues (cont.).

PDBID	Resolution (Å)	Number of residues	χ_{Score}	χ_{Score} percentile
1TIO	1.93	226	0.32	59.5
1TLD	1.5	226	0.71	72.9
1TNG	1.8	232	-1.29	11.9
1TNH	1.8	232	-0.61	30.4
1TNI	1.9	232	-0.88	22.1
1TNJ	1.8	232	-0.88	22.0
1TNK	1.8	232	-1.13	15.4
1TNL	1.9	232	-1.02	18.1
1TPO	1.7	226	-0.73	26.6
1TPP	1.4	226	-1.3	11.7
1TRY	1.55	227	0.63	70.0
1TX7	1.75	226	-1.29	11.9
1TX8	1.7	226	0.85	77.0
1TYN	2	226	0.23	56.7
1UTJ	1.83	246	0.71	72.7
1UTK	1.53	246	-0.86	22.7
1UTL	1.7	246	0.92	79.0
1UTM	1.5	246	-0.5	34.3
1UTN	1.15	247	-0.76	25.8
1UTO	1.15	247	0.65	70.8
1UTP	1.3	247	-0.74	26.1
1UTQ	1.15	247	-0.64	29.5
1V2J	1.9	226	-0.6	30.7
1V2K	2	226	0.22	56.2
1V2L	1.6	226	0.79	75.2
1V2M	1.65	226	0.93	79.4
1V2N	1.8	226	0.75	74.0

Table S7 Summary of trypsin models used for the detection of conserved strained residues (cont.).

PDBID	Resolution (Å)	Number of residues	χ_{Score}	χ_{Score} percentile
1V2O	1.62	226	0.8	75.7
1V2P	1.92	226	0.24	56.9
1V2Q	2.3	226	0.48	65.1
1V2R	1.7	226	0.66	71.1
1V2S	1.72	226	0.35	60.6
1V2T	1.9	226	0.25	57.2
1V2U	1.8	226	0.23	56.6
1V2V	1.8	226	-0.3	41.1
1V2W	1.75	226	0.48	65.2
1XUG	1.5	226	-1.44	9.2
1XUI	1.5	226	-1.58	7.1
1XUJ	1.92	226	-1.78	4.7
1XUK	1.8	226	-1.92	3.5
1XVO	0.84	227	0.79	75.2
1Y3U	1.8	226	-0.11	47.2
1Y3V	1.6	226	0.39	61.9
1Y3W	1.8	226	0.11	52.8
1Y3X	1.7	226	0.26	57.6
1Y3Y	1.75	226	0.48	65.1
1Y59	1.2	226	0.54	67.1
1Y5A	1.4	226	-0.4	37.8
1Y5B	1.65	226	0.64	70.5
1Y5U	1.6	226	0.68	71.7
1YP9	2.1	226	-0.43	36.5
1YYY	2.1	226	-1.86	4.0
1ZZZ	1.9	240	-1.86	4.0
2A31	1.25	226	-0.21	44.0

Table S7 Summary of trypsin models used for the detection of conserved strained residues (cont.).

PDBID	Resolution (Å)	Number of residues	χ_{Score}	χ_{Score} percentile
2A32	1.5	226	-0.33	40.0
2A7H	2.1	226	-0.99	18.9
2AH4	1.13	226	0.64	70.4
2AYW	0.97	226	0.23	56.5
2BLV	1.2	226	-1.09	16.3
2BLW	1.2	226	-1.1	16.1
2BY5	1.3	247	0.4	62.2
2BY6	1.3	247	0.56	67.9
2BY7	1.3	247	0.53	66.7
2BY8	1.3	247	0.41	62.7
2BY9	1.3	247	0.53	66.6
2BYA	1.3	247	0.52	66.3
2BZA	1.9	226	0.48	65.2
2D8W	2	226	-0.74	26.3
2EEK	1.85	223	-0.17	45.5
2FMJ	1.65	225	0.17	54.7
2FX4	1.65	226	-1.58	7.0
2FX6	1.57	226	-1.26	12.6
2G51	1.84	227	0.16	54.3
2G52	1.84	227	-0.53	33.2
2G55	1.82	226	0.47	64.8
2G5N	1.51	226	0.5	65.7
2G5V	1.45	226	0.39	61.9
2G8T	1.41	226	0.57	68.1
2OTV	1.56	226	0.44	63.7
2OXS	1.32	226	0.49	65.6
2PTN	1.55	226	-0.38	38.5

Table S7 Summary of trypsin models used for the detection of conserved strained residues (cont.).

PDBID	Resolution (Å)	Number of residues	χ_{Score}	χ_{Score} percentile
2TBS	1.8	225	-0.15	46.0
2TGA	1.8	232	-0.25	42.9
2TGT	1.7	232	-1.13	15.5
2TIO	1.93	226	0.91	78.9
2ZDK	1.67	226	-0.48	34.8
2ZDL	1.8	226	-0.74	26.4
2ZDM	1.93	226	-0.76	25.5
2ZDN	1.98	226	-0.58	31.6
2ZFS	1.51	226	0.85	77.0
2ZFT	1.76	226	0.66	71.1
2ZHD	1.94	226	-0.59	31.2
2ZPS	1.55	225	-0.2	44.3
2ZQ1	1.68	226	0.58	68.3
2ZQ2	1.4	226	-0.6	20.9
3A7T	1.75	226	-0.45	36.0
3A7V	1.75	226	-0.49	34.7
3A7W	1.75	226	-0.38	38.4
3A7X	1.75	226	0.42	63.1
3A7Y	1.81	226	-0.48	35.0
3A7Z	1.8	226	-0.2	44.4
3A80	1.75	226	0.49	65.4
3A81	1.78	226	-0.36	38.9
3A82	1.75	226	0.48	65.1
3A83	1.78	226	-0.41	37.2
3A84	1.75	226	0.49	65.3
3A85	1.75	226	0.4	62.3
3A86	1.75	226	-0.26	42.3

Table S7 Summary of trypsin models used for the detection of conserved strained residues (cont.).

PDBID	Resolution (Å)	Number of residues	χ_{Score}	χ_{Score} percentile
3A87	1.75	226	-0.47	35.4
3A88	1.75	226	-0.42	36.9
3A89	1.8	226	0.41	62.6
3A8A	1.4	226	0.23	56.4
3A8B	1.75	226	0.41	62.5
3A8C	1.85	226	0.42	63.1
3A8D	1.75	226	0.4	62.5
3AAS	1.75	226	0.21	56.0
3AAU	1.8	226	0.38	61.7
3ATI	1.71	226	-0.46	35.8
3ATK	1.74	226	0.61	69.4
3ATL	1.74	226	-0.36	39.2
3ATM	1.72	226	-0.44	36.2
3GY2	1.57	226	-0.79	24.7
3GY3	1.7	226	-0.66	29.0
3GY4	1.55	226	0.35	60.5
3GY5	1.57	226	-1.28	12.0
3GY6	1.7	226	-0.35	39.6
3GY7	1.55	226	0.74	73.9
3GY8	1.75	226	-0.55	32.7
3I77	2.1	233	-0.24	43.2
3ITI	1.55	226	-0.7	27.7
3LJJ	1.55	226	-0.73	26.4
3LJO	1.5	226	-0.41	37.5
3M35	2.2	226	0.34	60.1
3MFJ	0.8	226	0.43	63.5
3MI4	0.8	226	-0.32	40.5

Table S7 Summary of trypsin models used for the detection of conserved strained residues (cont.).

PDBID	Resolution (Å)	Number of residues	χ_{Score}	χ_{Score} percentile
3NK8	1.15	226	-0.44	36.2
3NKK	1.12	226	0.48	65.0
3PLB	1.18	226	1.46	91.1
3PLK	1.53	226	0.64	70.6
3PLP	1.63	226	0.7	72.5
3PM3	1.53	226	-0.51	34.0
3PMJ	1.45	226	-0.61	30.5
3PTB	1.7	226	-0.75	26.1
3PTN	1.7	226	-0.25	42.9
3PWB	1.63	226	1.18	85.7
3PWC	1.6	226	0.85	77.2
3PYH	2	226	-0.81	24.0
3Q00	1.7	226	0.69	72.0
3QK1	2.08	232	-1.03	17.9
3RXA	1.7	226	-0.39	37.9
3RXB	1.7	226	-0.56	32.2
3RXC	1.7	226	-0.31	40.6
3RXD	1.7	226	-0.47	35.2
3RXE	1.7	226	-0.3	41.3
3RXF	1.7	226	-0.32	40.4
3RXG	1.7	226	-0.46	35.4
3RXH	1.7	226	-0.4	37.7
3RXI	1.6	226	-0.45	35.9
3RXJ	1.7	226	-0.34	39.8
3RXK	1.6	226	-0.37	38.7
3RXL	1.7	226	-0.34	39.8
3RXM	1.7	226	-0.48	35.0

Table S7 Summary of trypsin models used for the detection of conserved strained residues (cont.).

PDBID	Resolution (Å)	Number of residues	χ_{Score}	χ_{Score} percentile
3RXO	1.6	226	-0.45	35.8
3RXP	1.6	226	-0.41	37.4
3RXQ	1.68	226	0.45	64.0
3RXR	1.72	226	0.52	66.3
3RXS	1.74	226	0.45	64.1
3RXT	1.7	226	-0.52	33.6
3RXU	1.68	226	0.43	63.4
3RXV	1.7	226	-0.4	37.6
3T25	1.7	226	0.43	63.4
3T26	1.7	226	-0.54	32.8
3T27	1.95	226	-0.79	24.7
3T28	2.8	226	-1.76	5.0
3T29	1.75	226	0.49	65.3
3UNQ	1.62	226	-1.22	13.3
3UNR	1.2	226	-0.53	33.3
3UNS	1.8	226	0.64	70.4
3UOP	1.69	226	-0.42	36.9
3UPE	1.54	226	-0.49	34.7
3UQO	1.8	226	-0.55	32.4
3UQV	2.4	226	-1.33	11.2
3UWI	1.43	226	0.71	72.7
3V0X	1.9	226	0.52	66.5
3V12	1.8	226	-0.49	34.6
3V13	1.63	226	-0.78	25.1
3VPK	1.94	226	-0.28	41.6
4AB8	1.6	226	-1.37	10.4
4AB9	1.2	226	-1.74	5.1

Table S7 Summary of trypsin models used for the detection of conserved strained residues (cont.).

PDBID	Resolution (Å)	Number of residues	χ_{Score}	χ_{Score} percentile
4ABA	1.25	226	-2	2.9
4ABB	1.25	226	-2.14	2.1
4ABE	1.3	226	-1.96	3.2
4ABF	1.3	226	-1.87	3.9
4ABG	1.52	226	-0.63	29.8
4ABH	1.25	226	-1.41	9.7
4I8G	0.8	226	-1.78	4.7
4I8H	0.75	226	-1.4	9.9
4I8J	0.87	226	-1.9	3.7
4I8K	0.85	226	-0.82	23.9
4I8L	0.87	226	-1.09	16.4
4M7G	0.81	230	2.42	99.0
4MTB	1.22	226	0.71	72.8
4NCY	1.42	226	-0.53	33.3
4NIV	1	226	0.68	71.7
4NIW	1.31	226	-0.14	46.3
4NIX	1.3	226	-0.35	39.5
4TPY	1.3	226	-0.59	31.2
4YTA	1.2	226	-0.53	33.3
5EG4	1.32	226	0.18	55.1
5F6M	1.1	226	-1.7	5.6
5FXL	1.78	250	-0.77	25.4
5JYI	1.91	250	0.76	74.2
5LGO	1.12	226	0.88	77.9
5LH8	1.54	226	-1.9	3.7
5PTP	1.34	226	-0.44	36.3
