# Supplementary material for: A distance geometry-based description and validation of protein main-chain conformation
Source: IUCrJ. 2017 Aug 8;4(Pt 5):657–70. doi: 10.1107/S2052252517008466 (PMC5619857; doi:10.1107/S2052252517008466)
Supplement: Supplementary file 2 [file m-04-00657-sup2.pdf]

# IUCrJ

**Volume 4 (2017)**


**Supporting information for article:**

## A distance geometry-based description and validation of protein main-chain conformation

**Joana Pereira and Victor S. Lamzin**

# Supplementary videos

**Figure S1** Conformational transition of the dipeptide units along the DipSpace *pc1* axis, demonstrating their ‘extension’.

**Figure S2** Conformational transition of the dipeptide units along the DipSpace *pc2* axis, demonstrating their ‘twist’.

**Figure S3** Conformational transition of the dipeptide units along the DipSpace *pc3* axis, demonstrating their ‘bending’.
